# Models for preclinical studies in aging-related disorders: One is not for all

**Published:** 2016-01-31

**Authors:** Gaetano Santulli, Consuelo Borras, Jean Bousquet, Laura Calzà, Antonio Cano, Maddalena Illario, Claudio Franceschi, Giuseppe Liotta, Marcello Maggio, William D. Molloy, Nunzia Montuori, Rónán O’Caoimh, Francesc Orfila, Amelia P. Rauter, Aurelia Santoro, Guido Iaccarino

**Affiliations:** 1 Department of Physiology and Cellular Biophysics, Columbia University Medical Center; College of Physicians & Surgeons, New York, USA;; 2 Department of Physiology, University of Valencia /INCLIVA, Valencia, Spain;; 3 MACVIA-LR, European Innovation Partnership on Active and Healthy Ageing Reference Site, University Hospital of Montpellier, France;; 4 INSERM, VIMA : Ageing and chronic diseases. Epidemiological and public health approaches, Paris, France; 5 Université Versailles St-Quentin-en-Yvelines, France;; 6 Health Sciences and Technologies - Interdepartmental Center for Industrial Research (HST-ICIR) University of Bologna;; 7 Department of Pediatrics, Obstetrics and Gynecology, University of Valencia/INCLIVA, Valencia, Spain;; 8 Department of Translational Medical Sciences, Federico II University, and R&D Unit, Federico II University Hospital;; 9 IRCCS Institute of Neurological Sciences, Bologna;; 10 National Research Council of Italy, CNR, Institute for Organic Synthesis and Photoreactivity (ISOF) and Institute of Molecular Genetics, Bologna, Italy;; 11 Dept. of Experimental, Diagnostic and Specialty Medicine (DIMES), University of Bologna, Bologna, Italy;; 12 Department of Biomedicine and Prevention “Tor Vergata” University of Rome, Italy;; 13 Department of Clinical and Experimental Medicine, University of Parma; University Hospital of Parma; 14 Centre for Gerontology and Rehabilitation, University College Cork, Ireland;; 15 Health Research Board, Clinical Research Facility Galway, National University of Ireland, Galway, Ireland;; 16 Institut Universitari d’Investigació en Atenció Primària Jordi Gol (IDIAP Jordi Gol), Barcelona, Spain;; 17 Departamento de Quimica e Bioquímica, Universidade de Lisboa, Portugal;; 18 Department of Medicine and Surgery, University of Salerno, Italy.

**Keywords:** aging, animal models, rodents, swine, cardiovascular medicine, preclinical studies, frailty, multimorbidity

## Abstract

Preclinical studies are essentially based on animal models of a particular disease. The primary purpose of preclinical efficacy studies is to support generalization of treatment–effect relationships to human subjects. Researchers aim to demonstrate a causal relationship between an investigational agent and a disease-related phenotype in such models. Numerous factors can muddle reliable inferences about such cause-effect relationships, including biased outcome assessment due to experimenter expectations. For instance, responses in a particular inbred mouse might be specific to the strain, limiting generalizability. Selecting well-justified and widely acknowledged model systems represents the best start in designing preclinical studies, especially to overcome any potential bias related to the model itself. This is particularly true in the research that focuses on aging, which carries unique challenges, mainly attributable to the fact that our already long lifespan makes designing experiments that use people as subjects extremely difficult and largely impractical.

## I. INTRODUCTION

In the European Union the number of people aged >75 years is projected to double by the year 2060, thus comprising 20% of the total population (1,2). These changes will lead to an increase of 20 to 40% of the costs necessary to maintain the existing quality of healthcare services. The European Commission is promoting through the Innovative Partnership on Active and Healthy Aging the discussion between multiple stakeholders on what are going to be the priorities for tackling this new societal challenge (http://ec.europa.eu/research/innovation-union/index_en.cfm?section=active-healthy-ageing).

Education and research are going to be pivotal for the identification of the mechanisms of healthy aging and to prevent conditions that mine an active and healthy during a life course. In particular, multimorbidity is almost constant in the oldest old and has adverse consequences such as higher mortality, poorer quality of life and reduced functional status (3). Geriatricians commonly use the term “frailty” to describe a biologic syndrome of decreased reserve, resilience and resistance to stressors, resulting from cumulative declines across multiple physiologic systems, causing vulnerability to adverse outcomes. Nevertheless, aging is a complex process that involves both a decline in the function of an organism and a greater risk of disorders associated with growing older (4, 5). Researchers have identified animal genes that influence lifespan, some of which modify the aging process as a whole, others which act by increasing or decreasing age-related illnesses (6, 7). Several species of animals have figured prominently in aging research (8). Such research has mostly focused on the genetic basis of aging, and on learning more about pathophysiological pathways that regulate the rate of aging and set the stage for age-related disorders.

## II. PRIMITIVE ORGANISMS

The yeast, a single-celled fungus (*Saccharomyces cerevisiae*), is one of the most intensively studied eukaryotic model organisms in molecular and cell biology (9), much like *Escherichia coli* as the model bacterium (10). It serves as a useful model because it shares a lot in common with animals, at least at the cellular and genetic levels (11). Due to its short lifespan, yeast represents an ideal model in aging research (12). Mammals have some genes that correspond to some of those associated with longer life in yeast, and an understanding of the workings of the yeast genes could foster our understanding of the mammalian ones.

Equally important, the nematode *Caenorhabditis elegans* is a roundworm with a ∼20-day lifespan (13). Hitherto, more than 400 genes that extend lifespan in roundworms have been described (14). The roundworm genes that seem to confer increased longevity do so by supporting resistance to different forms of stress, including oxidative damage, bacterial infections and high temperatures (15). The correlation between the existence of roundworm genes and their mammalian counterparts indicates that *C. elegans* will continue to be a valuable animal model for the study of aging (13).

The fruit fly *Drosophila melanogaster* is another favorite subject for studies on longevity. Mutant versions of a particular gene Indy, short for “I’m Not Dead Yet”, have been shown to double the fruit flies’ average lifespan (16). The protein encoded by the Indy gene is closely related to a human protein active in energy production (17). Since the fruit fly has genes such as Indy that produce proteins very similar to human proteins, it makes a tremendous model organism for aging research.

## III. NON-HUMAN PRIMATES

The discovery that fruit flies and roundworms carry genes that affect their longevity is enthusing, particularly because many of those genes have human counterparts. However, the complexity of human physiology cannot be replicated in simple organisms such as fruit flies and roundworms. In this sense, non-human primates occupy a special niche as models for health and disease because, with their close phylogenetic relationship to humans (18), they often closely mirror the physiological processes that take place in humans. Indeed, our DNA is very similar to that of non-human primates, including monkeys, apes, and chimpanzees. Several experiments into aging and longevity using primate models such as rhesus and squirrel monkeys are currently ongoing in studies of neurobiology, skeletal deterioration, reproductive aging, and other age-related disorders (19). Rhesus monkeys are particularly useful because the rate of aging in rhesus monkeys is three times as fast as the rate in humans. In a recent study, rhesus monkeys were given 30% fewer calories compared with control animals over a 23-year period (20). Researchers found no increase in longevity for the calorie-restricted animals, but they confirmed, however, that eating less may improve health by delaying the onset of diseases such as diabetes, cancer and cardiovascular disease (20). The common marmoset (Callithrix jacchus) is poised to become a standard non-human primate aging model (21). With an average lifespan of 5 to 7 years and a maximum lifespan of 16–17 years, marmosets are the shortest-lived anthropoid primates (22). They display age-related changes in pathologies that mirror those seen in humans, such as cancer, amyloidosis, diabetes, and chronic renal disease (19).

## IV. SMALL RODENTS

Mice and rats represent the animal models of choice for scientists interested in aging for several reasons. First, they are mammals, thus more closely related to us than yeast, worms or flies. Second, their relatively small size and short lifespan make them easier to study than long-lived animals (1)(23–26). Much of the excitement in recent aging research has come from discoveries that aging can be postponed in mice (27) or rats (28) by very low calorie diets, and by discoveries of mutant genes that can extend lifespan by as much as 50 percent. Studies of these slow-aging rodents may prove helpful in the development of treatments that could prevent late-life disorders by mimicking the effects seen in the animals. The growing interest in rodents’ aging has been intensely stimulated by the sequencing of mouse and human genomes and by the realization that most human genetic diseases can be modeled by changes in equivalent genes in these small animals. Through targeted genetic manipulation, researchers have created genetic lines of mice that model Alzheimer’s disease, Werner’s syndrome (premature aging), diabetes, atherosclerosis, immune dysfunctions, oxidative stress, musculoskeletal disorders, and other medical conditions associated with aging (29–31). All these mouse models are actually providing novel insights into aging mechanisms.

## V. SWINE MODELS

Owing to their genomic, anatomical and overall physiological resemblance to humans (32), swine models have been extensively used in biomedical research (33), especially in studies about cardiovascular function and bone physiology. Indeed, porcine hearts exhibit coronary artery anatomy and gross anatomic structure very similar to that of humans and have been the subject of several translational studies (34). Pigs and humans are also mostly similar with respect to bone composition, microstructure and remodeling (35). Generally, there are unique advantages to the use of swine models in translational research, given that they share with humans similar anatomic and physiologic characteristics also in the urinary, integumentary, and digestive systems (36, 37). Pigs have been used as models of myocardial ischemia in the setting of graduated treadmill exercise training and increasing oxygen demand (38). However, a perceived difficulty of using the pig model of myocardial infarction is a predisposition for refractory arrhythmogenesis (39). Several strategies have been described to obviate this issue, including aggressive airway protection and ventilatory management, electrolyte supplementation and antiarrhythmic administration (40).

Porcine coronary arteries are also considered an excellent model to assess safety and efficacy of devices under development for intracoronary applications, including novel imaging and stent technologies (41). Indeed, cardiac catheterization and coronary intervention in the pig are similar in many ways to the human. However, other major limitations of normal swine models include a large size and a low propensity to atherosclerosis even with prolonged feeding of high fat diets (42). To overcome these issues, the use of genetically modified miniature (mini-pigs) has emerged over recent years, with the creation of models of hypercholesterolemia, atherosclerosis and metabolic syndrome (37, 43). Thus, it is likely that mini-pigs will become an increasingly important animal model for research and pharmaceutical development applications.

## VI. ANIMAL MODELS OF COMMON AGE-RELATED DISORDERS

### Bone disease

Age-related bone loss is a multifactorial skeletal disease, characterized by disruption of the microarchitectural structure of bone tissue and reduction in bone mass, resulting in loss of mechanical strength and increased risk of fracture (44). Such a disorder might be localized or involve the entire skeleton. In the European Union osteoporosis is a leading cause of mortality and morbidity in older adults, representing a key factor in the high cost of medical care (45).

Many therapeutic advances in the management of osteoporosis were studied first in diverse animal models and then entered clinical practice (46). Animal models that have been used in the past include non-human primates, dogs, cats, sheep, rabbits, mini-pigs, guinea pigs and other small rodents, all of which have advantages and disadvantages (47). Among these, the laboratory rat is one of the preferred animals for most researchers. Its skeleton has been extensively studied, and although there are several limitations to its similarity to the human condition, these can be overcome through detailed knowledge of its specific traits or with certain techniques. The rat has been used in a number of experimental protocols leading to bone loss, including hormonal interventions (e.g. ovariectomy, parathyroidectomy, hypophysectomy, orchidectomy), immobilization, and dietary manipulations (48). Rat osteopenia due to age, ovariectomy (in the female rat), and immobilization bears a strong resemblance to human osteopenia, both in its anatomical features as well as in the transitional and steady states of the bone dynamics. In particular, Wistar rats display progressive loss of bone density both at trabecular and cortical sites along with cortical thinning after 12 months of age (49).

A potential drawback to the use of rat models for osteoporosis is the lack of Haversian remodeling (osteon). In humans, increased Haversian remodeling in the skeleton is the main cause of cortical porosity. In the rat skeleton, cortical bone gain occurs in the periosteum, and cortical bone is lost at the endosteum (46). Larger animal models such as dogs and primates are generally considered more appropriate for the study of Haversian remodeling. However, the species-specific traits of osteoporosis in dogs (ethical dilemmas, inappropriate model for postmenopausal osteoporosis, high cost of maintenance,) and primates (high cost of acquisition and maintenance, reduced availability in experimental centers, ethical dilemmas) definitively limit their use.

Trabecular and cortical bone deficits with aging have been also described in female C57/BL6 mice (50). Of interest, female mice do not have the equivalent of a menopause: they simply undergo reproductive senescence, becoming essentially acyclic by 11 to 16 months of age (51). A recent study dissociated the effects of aging per se versus those of age-related estrogen deficiency in mice, demonstrating that by maintaining physiologic levels of estrogen in aging female mice can prevent cortical bone loss, trabecular bone loss over the lifespan is mostly independent of endogenous estrogen levels (50).

The classic mouse model of senescence acceleration and age-associated disorders is the “senescence accelerated mouse” (SAM) mouse. It has been under development since 1970 through the selective inbreeding of AKR/J by a research team at Kyoto University. It consists of 14 senescent-prone inbred strains (SAMP) and 4 senescence-resistant inbred strains (SAMR). In particular, the SAMP6 strain was established as a model for senile osteoporosis characterized by low peak bone mass at their maturation (52). The low bone mass in these mice is polygenetically determined, a situation akin to osteoporosis in humans. Intriguingly, in vitro analysis confirmed that bone marrow–derived stem cells isolated from SAMP6 mice had a markedly reduced osteogenic capacity (48).

### Cardiovascular disease

The use of animal models has been crucial in the progression of developing new clinical therapeutics for cardiovascular disorders. One of the greatest successes in translated therapies of heart failure has been given by the β–adrenergic receptor antagonists (2, 53–56). The development of such a therapy occurred only after extensive animal modelling work at several institutions. Additionally, basic science and clinical research findings were integrated both from bench-to-bedside and bedside-to-bench to help develop effective clinical therapies. Thus, the success of this therapy highlights the importance of carefully designed animal research.

Rodents have been largely used as model of cardiovascular disorders. For example, the spontaneously hypertensive rat (SHR) is a well-studied animal model of human essential hypertension (57, 58), a very common disease among older adults (59). This inbred strain was simply developed by selective breeding of the Wistar-Kyoto (WKY) stock for higher blood pressure. Rodents are also used as models of myocardial infarction, restenosis post angioplasty, and heart failure (60–62). Myocardial infarction is usually achieved both in rats and mice by surgical ligation of a coronary artery (63). Heart failure can be obtained through means of myocardial infarction, cryo-injury, inducing a pressure overload after pharmacological challenge with adrenergic agonists or by ascending aortic banding (64, 65).

Although numerous discoveries regarding myocardial dysfunction have been made with the use of murine or canine models, several confounding factors including collateral coronary circulation contributed to a transition to alternative animal species, such as sheep and pigs in the study of myocardial ischemia (66). Consistent coronary arterial anatomy, lack of preformed collateral vessels, and the ability to create infarctions of predictable size and location make both pigs and sheep reasonable choices for studying myocardial ischemia and post-infarction ventricular remodeling.

The pig epicardial coronary artery distribution closely resembles that of humans, although with fewer collateral vessels. The left main coronary artery generally bifurcates early into a left anterior descending and circumflex coronary arteries. These vessels are of similar diameter to those in the human (2.0 – 4.0 mm). The right coronary artery is also usually of similar diameter to the human, although it is less often dominant (supplying the posterior cardiac surface) than in the human (67). The basic cardiac hemodynamic parameters and platelet characteristics are also comparable, though there are important differences between the porcine and human coagulation and fibrinolytic systems. Porcine models of myocardial infarction have been used to study infarct expansion and ventricular remodeling in the post-infarction setting. For instance, the induction of myocardial infarction in adult pigs facilitated placement of radiopaque markers, in order to reliably quantify the progressive infarct expansion, thus creating a model suitable for studying pharmacological therapies aimed at attenuation of infarct expansion (68). Recent studies have examined the impact of porcine stem cell transplantation on myocardial function in swine models of cardiac infarction, finding that stem cells were able to improve contractile function in infarcted and border-zone myocardium (69). Angiogenic growth factors have also been delivered to infarcted myocardium in a pig model, revealing re-establishment of stable collateral networks and improved myocardial perfusion (70).

### Aging lung

The aging lung is characterized by notable changes in both structure and function. Morphologic changes in the respiratory system consist of significant reduction in the elastic recoil of the lung, greater chest wall rigidity, and loss of power in the respiratory muscles (2, 71). Early investigations of the regulation of airway function and its potential relationship to asthma and chronic obstructive pulmonary disease (COPD) employed large animals including dogs, cats and non-human primates. The detailed study of respiratory system mechanics was easily performed in such models whereas equipment to study smaller animals was not generally available (72). In the past 30 years the techniques available for small rodents have improved substantially and have undergone extensive testing. Non-invasive techniques for the mouse have been developed to facilitate the assessment of pulmonary function but are associated with a substantial degree of uncertainty. Indeed, studies in murine models of aging, including the SAM mouse (73), the klotho mouse (74) and the Senescence Marker Protein-30 (SMP30) knock-out mouse (75) provided controversial results (76). Though, the measurement of pulmonary function in the mouse is somewhat more difficult to make than in the rat. The Brown Norway rat represents the most suitable strain for the study of allergen-induced airway reactions (which have many features in common with human asthma), especially given its high IgE levels (77). On the other hand, the Fisher 344 rat represents an excellent model to study COPD, mainly due to its innate airway responsiveness (78).

### Neurodegenerative disease

Neurodegenerative disorders are characterized by a progressive degeneration of neurons in specific locations of the central nervous system. Ideally, animal models of neurodegenerative disease should reproduce all of the changes specific to a given disease. Several animal models have been developed to test neuroprotective strategies in neurodegenerative disorders. However, most of them are poorly predictive of an effect in patients (79), mainly because the etiology and the clinical manifestations may differ from one patient to another.

Most of the models of Parkinson’s disease are based on the use of a neurotoxin that mimics the effect of environmental toxins or reproduces the biochemical changes seen in such a disease (80). Animal models of Alzheimer’s disease have relied on the utilization of genetic mutations associated with familial forms of Alzheimer’s disease. Transgenic mice overproducing mutant amyloid precursor protein develop a pathology that is structurally similar to that found in the human brain (81). These transgenic models exhibit memory impairments. Interestingly, the cognitive deficits occur earlier than the appearance of extracellular plaques. This observation led to a search for earlier pathological hallmarks that could be mediating cognitive decline.

Unfortunately, most of the existing models do not reproduce the full spectrum of the lesions and symptoms seen in humans. Animal models of neurodegenerative disease have existed since the late 1950s when reserpine was used to obtain a Parkinson’s-like phenotype in animals (82). For the next three decades animal models mainly consisted of using different drugs and toxins to create lesions in specific brain regions to mimic various diseases. These models played an essential role in the elucidation of basic functional neuroanatomy and circuitry. However, they did not help us understand the underlying disease mechanisms.

The significance of animal models has changed drastically in the last years, mainly because the identification of disease genes has allowed the creation of new models (83). Transgenic animals expressing the human mutant genes have been created. The insights generated by studying these animals have revolutionized our understanding of these complex human disorders. Molecular twists have been used to create transgenic models in which a particular gene can be turned on and off and even selectively expressed in only certain neurons. Knock-in and knock-out models of genetic defects have also been made. These animal models have proved to be powerful tools for studying the biology underlying the disease process (83, 84). Normal and abnormal protein function can be studied in vivo to identify protein interactors and elucidate the involved molecular pathways. Genetic screens in invertebrate models of neurodegenerative disease (including yeast) enable the identification of suppressors or enhancers that can modify the disease phenotype.

As new technologies have been developed over the decades, their application to animal models has led to important discoveries. For instance, laser capture microdissection of transgenic animal models and even postmortem human brain samples give us the capacity to analyze individual neurons quickly and efficiently (85). Most recently, in order to study more sophisticated transgenic models of neurodegenerative disorders, sheep, pig and primate models have been made. Generally, non-human primates are preferred to rodents when, for example, a new therapeutic compound has to be tested before clinical trials. However, non-human primates as models for the study of neurodegenerative disorders also present some limitations, including the great inter-individual variability on the response to a particular treatment and the difference in the symptoms of a particular disease when compared to humans (86).

## VII. MULTI-MORBIDITY AND FRAILTY

As discussed above, preclinical models of aging are certainly needed to dissect the molecular mechanisms underlying the decline in overall physical performance observed in humans (87). In this sense it is important to highlight the concept of multi-morbidity in older adults: more than 70% of people over 65 years have two or more chronic conditions (88) including arthritis, diabetes mellitus, cancer, heart disease, and stroke. Thus, as stated in a recent Nature Commentary (89), the issues of old age come as a package.

Frailty in older adults has been defined as a clinical syndrome characterized by skeletal muscle weakness, increased inflammation, and multi-systemic decline; it is also associated with a high risk of adverse health outcomes such as disability and mortality (90). Albeit only recently characterized, frailty is an important geriatric syndrome consisting of a reduced physiologic reserve that increases vulnerability to dependency and/or death and is globally estimated to afflict up to 27% of the elderly (91). Despite recent advances in frailty research in human cohorts, the mechanisms that mediate musculoskeletal decline and adverse outcomes in frailty remain unclear. The development of animal models that approximate human frailty is therefore necessary to facilitate etiologic and treatment-focused frailty research. Parks and colleagues reported a first attempt of an animal frailty scale based on the deficit accumulation model, which includes 31 variables involving activity levels, hemodynamic measures, body composition, and several metabolic parameters (92).

The latest animal models of frailty (93) represent a critical step in the right direction. For instance, the interleukin-10 knockout mouse has been proposed as a model for human frailty, since it develops an age-related decline in skeletal muscle strength compared to control mice (94). This genetically modified frail-mouse model also mimics the inflammation and weakness that often afflicts older people.

Frailty can also be modelled in naturally aging mice as a frailty-phenotype score, graded by such performance measures as grip strength and walking speed. Importantly, the work on frailty has mainly used male C57BL/6J mice. While this strain is generally used, complications may arise in the context of frailty as the strain is known to be particularly predisposed to cancer of the lymphatic and hematopoietic systems; researchers should take into account that the mechanisms that drive these cancers may also drive mouse frailty, potentially by different pathways than those that underlie human frailty. Ergo, more-sophisticated animal models of frailty should include a broad range of performance measures in order to properly represent the conditions observed in humans.

## VIII. CONCLUSION

Animal models have been and will continue to be essential in developing new clinical therapies. However, the road to successful translation is intricate and requires several careful considerations, including an appropriate choice of animal models, systematic experimental design, and integration of information from both the bench and the bedside.
